# Influence of Asymmetric Rolling Process on the Microstructure Properties of Bimetallic Sheet Metals

**DOI:** 10.3390/ma15062013

**Published:** 2022-03-09

**Authors:** Grzegorz Stradomski, Dariusz Rydz, Tomasz Garstka, Michał Pałęga, Tomasz Dyl, Arkadiusz Szarek, Justyna Łukomska Szarek, Tomasz Dembiczak

**Affiliations:** 1 Faculty of Production Engineering and Materials Technology, Czestochowa University of Technology, 19 Armii Krajowej Av., 42-201 Czestochowa, Poland; dariusz.rydz@pcz.pl (D.R.); tomasz.garstka@pcz.pl (T.G.); michal.palega@pcz.pl (M.P.); 2 Department of Marine Maintenance, Faculty of Marine Engineering, Gdynia Maritime University, Morska Street 81-87, 81-225 Gdynia, Poland; t.dyl@wm.umg.edu.pl; 3 Department of Technology and Automation, Faculty of Mechanical Engineering and Computer Science, Czestochowa University of Technology, 21 Armii Krajowej Av., 42-201 Czestochowa, Poland; arek@iop.pcz.pl; 4 Faculty of Management, Czestochowa University of Technology, 19 B Armii Krajowej Av., 42-201 Czestochowa, Poland; j.lukomska-szarek@pcz.pl; 5 Faculty of Science and Technology, Jan Dlugosz University in Czestochowa, Armii Krajowej Street 13/15, 42-200 Czestochowa, Poland; t.dembiczak@ujd.edu.pl

**Keywords:** asymmetric rolling (ASR), microstructure, plastic deformation, mechanical properties

## Abstract

This paper presents the results of research on the determination of the influence of kinetic asymmetry of work rolls on structural changes in hot-rolled bimetallic sheet metals. The tests were conducted on bimetallic samples composed of materials 10CrMo9-10 + X2CrNiMo17-12-2. The scope of the research included a comparative analysis for two cooling variants: I in water (freezing the structure immediately after rolling) and II for cooling in air. The research conducted showed that the introduction of asymmetric conditions to the rolling process results in a greater grain fragmentation in the so-called hard layer and does not have a negative effect on microstructural changes in the soft layer.

## 1. Introduction

**Figure 1:**
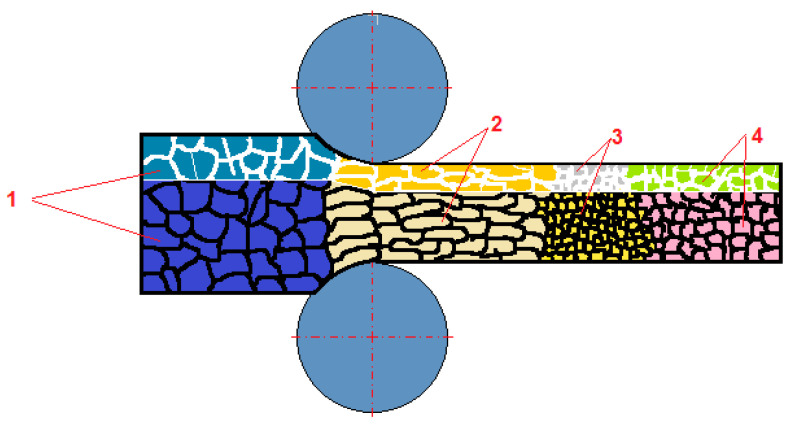
Scheme of grain size changes occurring in the structures of plastically deformed metals during hot rolling; 1, bimetal layers; 2, static recovery; 3, static recrystallization; 4, grain growth (based on [7,12,13,14,19,23]).

Nowadays, bimetallic materials have become more and more popular. This is caused mainly due to the need to improve the functional parameters of constructions (for example, in transport, lighter bodied cars are now standard) [1,2]. However, cars are not only the main field of interest for constructors using bimetals. Those materials are also present in power plants, shipbuilding, architecture, chemistry, and food productions [3,4,5]. There are many different possible technologies that can be used for both joining and final shaping. One of them is the rolling process. It consists in changing the shape of plastically processed materials as a result of the interaction of external forces. Both the geometrical dimensions of the rolled sheet metal and its internal structure change [6,7,8]. Metals and their alloys intended for further processing should be marked by a high plasticity, since hard or brittle materials fracture and get damaged under the influence of the applied pressure. The properties, which are responsible for the occurrence of hardening or its absence, have a decisive influence on the behavior of metals subjected to rolling [9,10,11]. The impact of the rolling process on structural changes of homogeneous sheet metals is relatively well-described in the literature [12,13,14]. However, the matter becomes more complicated in the case of bimetallic sheet metal rolling [15,16,17], where it is even imposed to use an asymmetric rolling process as a method of reducing the curvature of the two-layer band. Bimetal materials have separate properties that may cause differences in the course of the ongoing structural changes [18,19,20]. Based on the literature [19,20,21] and according to the authors of the publication, these changes can be presented according to the diagram (Figure 1). It was assumed that rolling is performed for optimal process conditions (i.e., ensuring obtaining a straight sheet metal, after leaving the rolling gap, and the evenest distribution of the total deformation into the layers of the bimetallic band) [22,23,24]. The preliminary assumptions made before the tests assumed that the introduced asymmetry of the circumferential rate of the work rolls would make it possible to obtain both a product with the desirable geometry and favorable mechanical properties of bimetallic materials. Depending on the type of material and deformation parameters, the effects of deformation strengthening can be removed by dynamic or static healing and (static, dynamic, and metadynamic) recrystallization [25,26,27]. During the rolling process, the phenomenon of dynamic recrystallization or dynamic recovery occurs during deformation [28,29,30,31,32].

In the case of rolling bimetallic sheet metals, the distribution of the total relative deformation into individual layers of the bimetallic sheet metal plays an important role in the course of structural changes [31,32,33,34]. It is very often observed during the rolling of double-layer sheet metals with large differences in deformation resistances that one of the layers is deformed only to a slight extent or not at all. This translates directly into the course of structural changes in this layer. It is highly probable that the phenomenon of recrystallization in this layer will not occur at all. As a result, after the rolling process, this layer may have worse mechanical properties than before the rolling process. Another problem with the uneven distribution of the deformation into the layers is the bending of the bimetallic band after it comes out of the rolling gap [11,18,20]. One of the methods of increasing the evenness of deformation of bimetallic sheet metals and obtaining a simple sheet metal is to introduce asymmetry of the peripheral speed of the work rolls (i.e., kinetic asymmetry) [17,25,30].

Based on the literature review, it can be seen that there is a lack of presented information about the determination of the impact of the asymmetry of the rolling process on the microstructural changes occurring in the layers of bimetallic sheets subjected to hot rolling. That was the main scope for the authors of this study to take up this issue, and the results of the conducted research are presented in the paper below. The presented results are the authors’ own work and are related to their earlier work presented in conferences and scientific papers.

### Asymmetry of Kinetic Velocity

Until recently, the use of asymmetry of the peripheral speed of work rolls resulted mainly from the necessity to eliminate the disadvantage, which is the curvature of double-layer sheet metals after leaving the deformation zone. This task is still not easy to solve, and solving it causes many problems. In recent years, as a result of the growing demand for this type of product, there have also been increased expectations as to the quality of flat bimetallic products. Therefore, it is necessary to carefully examine the influence of the joining process and the subsequent rolling process on the properties of finished products. In the joining process, the selection of the method depends in most cases on the quality of the obtained joint. Primarily, attention should be paid to the durability and strength of the joint, as well as the quantitative assessment of the intermetallic presence in the area of the joint. In the rolling process, plastic deformation is conducted so as to obtain both the desirable shape and mechanical properties of the finished product. In order to achieve these assumptions during the rolling process, it is necessary to determine the optimal conditions for its implementation. If the rolling process is conducted at the same velocities for most pairs of materials, deviation from the optimal parameters results in obtaining a product marked by an inconsistent shape (i.e., a bent sheet of metal) [11,18,20]. The process of rolling flat products requires anywhere from a few to over a dozen passes. The number of passages depends on the initial and final dimensions of the rolled bimetallic sheet metals. The curvature of the bimetallic band caused by the uneven elongation of the layers especially during first passes is a huge problem. This is mainly due to the lack of possibility to hold the band in the next pass. Traditional, symmetrical rolling of two-layer flat products cause bending due to the uneven deformation. The curvature (bending) value is influenced by many factors presented in the literature such as deformation resistance of the bimetal components or the conditions of the rolling process. That is why when talking about rolling of bimetallic sheet metals it is by definition an asymmetrical process. Well-made rolling process parameters control can lower those problems or even give possibility to eliminate it. Use of asymmetrical rolling process (ASR) with roll diameters or their rotational speeds can eliminate the effect of the bent band after coming out of the rolling gap. It can also be observed that making rolling process as an asymmetrical one affects the fragmentation of the microstructure of homogeneous products. The same or similar effect is observed during asymmetrical rolling process of bimetallic sheet; not only curvature control is possible but also grain size refinement occurs. For the final products, parameters grain size is one of the main factors.

The first mentions of the use of ASR processes were recorded in the first part of the XX century in the 1940s [32]. Initially, it was used in particular during rolling process of flat bimetallic products. The main aim to use of the asymmetrical rolling process of double-layer sheet metals resulted mainly from differences in the properties of bimetallic materials. The effect of this unevenness is to obtain a bent band after it comes out of the rolling gap. The value of this bent is influenced by many factors, including differences in the deformation resistance of the bimetal components or the conditions of the rolling process. Being mindful of obtaining a simple double-layer sheet metal, the asymmetry of the peripheral speed of the work rolls was used, as described by the equation [3,4,5,20]:

(1) $$a_{v}=\frac{V_{g}}{V_{d}},$$

where:

*V_g_*, *V_d_*—peripheral speeds of the upper and lower rolls

**Figure 2:**
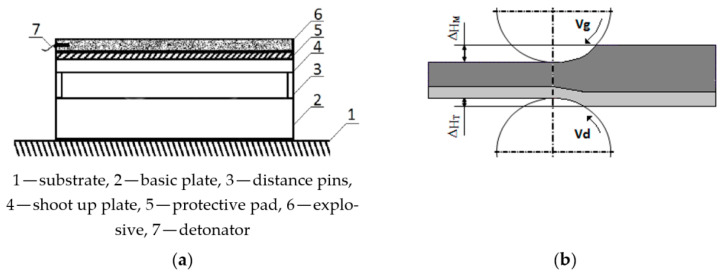
Stages of production of bimetallic plates, (**a**) combining by the method of explosive welding (**b**) scheme of asymmetrical rolling of bimetallic plates based on [3,17,19,29].

At the turn of the 20th century, the first mentions of the use of asymmetry to shape the structure of homogeneous materials were noted [9]. There are relatively many works related to modeling structural changes [7,23]. However, they mostly concern homogeneous materials. Therefore, the influence of the asymmetry of the peripheral velocity of the work rolls during the rolling of bimetallic sheet metals on the occurring structural changes was analyzed in this study. A comparative analysis was carried out on the microstructure after the first stage of producing bimetallic sheet metals (i.e., joining using the explosive welding method), and the microstructure after a symmetrical and asymmetrical rolling process (Figure 2).

On the basis of numerous works [3,17,19,22], it has been shown that the introduced asymmetry of the peripheral velocities of the work rolls enables an even flow of both layers of bimetal in the plane of exit from the rolling gap, and thus the possibility of such correction of the band shape to obtain a finished product with the shape provided for by the standards. The authors of works related to asymmetric rolling mainly focus on controlling the shape of the band and lowering the energy and strength parameters in comparison to the process conducted symmetrically, ignoring the important aspect related to the impact of the rolling process carried out in this way on the ongoing structural changes (particularly for bimetallic sheet metals).

## 2. Materials and Method

**Figure 3:**
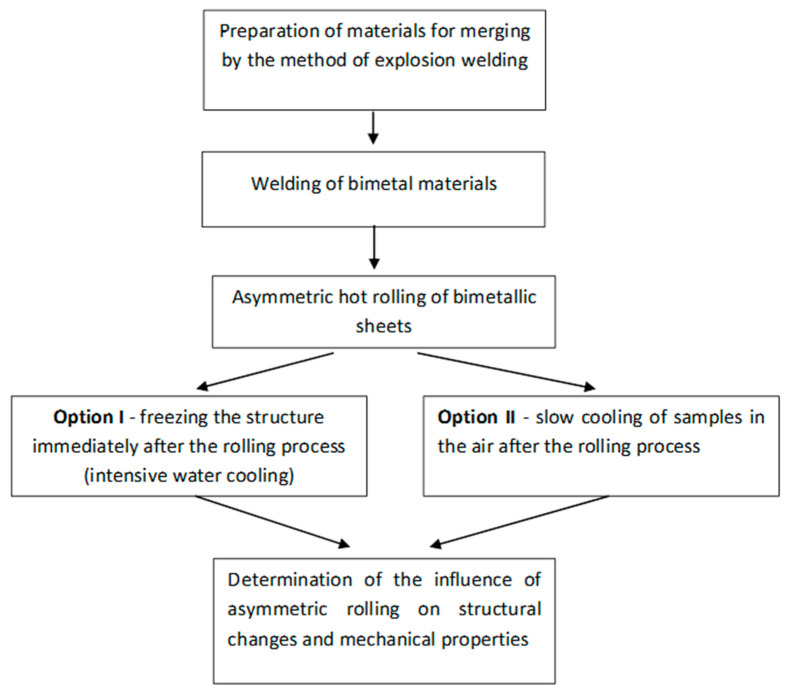
Scheme of research made in the work.

A comparative analysis was performed on the structure of bimetallic sheet metals after the rolling process with the same speed of the rolls and diverse speeds of the rolls, for which a simple bimetallic sheet metal was obtained. To determine the optimal value of the speed of the work rolls, the Forge 3D program was used, the calculations of which are based on the finite element method. The optimal conditions for the rolling process of bimetallic sheet metals composed of 10CrMo9-10 and X2CrNiMo17-12-2 steel were determined based on the computer simulations. The scheme of the presented research is shown in Figure 3. Purpose and scope of the conducted research.

**Figure 4:**
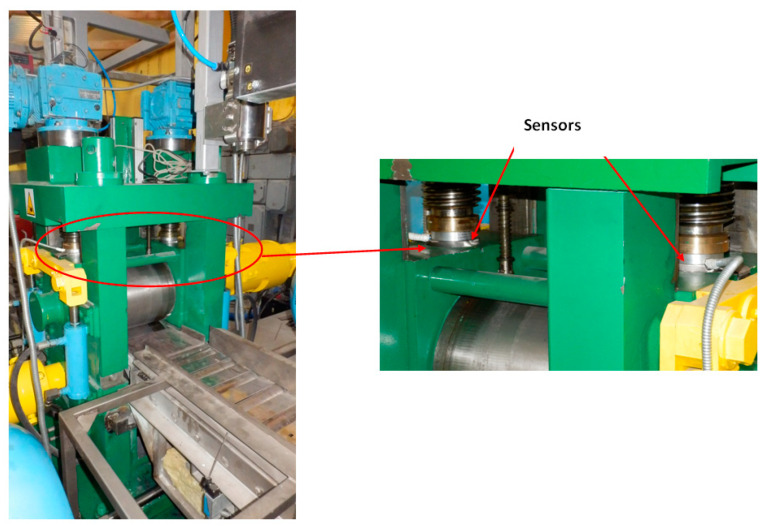
View of the laboratory rolling mill at mill DUO 300.

The next step was to conduct the rolling process on a semi-industrial rolling line (Figure 4), the main element of which was the DUO 300 rolling mill (DUO means two rolls rolling mill with roll diameter 300 mm). The rolling line is equipped with the measuring device of the rolling mill that enables the measurement of energy and strength parameters of the rolling process. The measurement was made directly by the sensors of the rolling force and moment, indirectly on the basis of signals received from frequency converters supplying the driving motors of the upper and lower roll. During the experimental tests, the metal pressure force on the rolls was recorded in the measuring system of the DUO-300 rolling mill, which was measured using two CL21 force transducers with a range of 250 kN, placed between the upper roll bearing cases and the adjusting screws.

**Figure 5:**
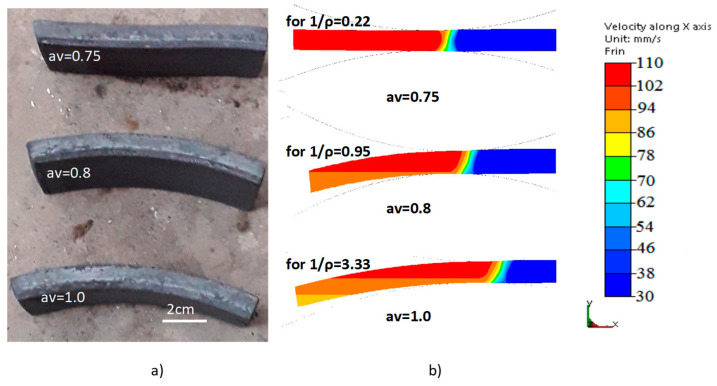
View of samples after asymmetric rolling (**a**) laboratory tests, (**b**) computer simulations.

The experimental tests were performed for bimetallic sheet metals composed of 10CrMo9-10 and X2CrNiMo17-12-2 steels with a thickness of 12 mm (where 8 mm was 10CrMo9-10 steel and 4 mm was X2CrNiMo17-12-2 steel), the chemical composition of which is presented in Table 1. The samples were heated in a chamber furnace to a temperature of approx. 1100 °C for 20 min. After heating, the samples were transported by roller table to the rolling mill. The rolling process was conducted at 1080 °C and then cooled with water to freeze the structure for variant I, and cooled in the air for variant II. The view of representative samples is shown in Figure 5. The research was conducted both for the process of symmetry of the peripheral velocity of the work rolls and for the numerically determined optimal conditions of the asymmetric rolling process for *a_v_* = 0.75 and crush ε = 0.18. Numerical modeling of the asymmetric rolling process was carried out in the Forge 3D program. Numerical studies were conducted within the scope of asymmetry coefficients *a_v_* = 1.0 to 0.75. The analysis covers both the curvature of double-layer sheet metals and the influence of the diversity of the peripheral velocities of the work rolls on the structural changes occurring in the bimetallic materials.

Initial numerical tests of the asymmetric rolling process were performed with the use of the *Forge*3D^®^ program [35]. The first stage of numerical research was to import the designed tools and input material, which form the objects of computer simulation. In order to obtain the most accurate results of numerical tests, a very important stage of design is the right refinement of the finite element mesh [36,37] on the surfaces of the object. A triangular mesh was used in this case. The *Forge*3D^®^ program enables the mesh concentration in selected areas (e.g., at corners, roundings, in place of complex shapes, and small dimensions by defining mesh concentration zones called “mesh-boxes”). Computer simulations were carried out for the rolling of bimetallic sheet metals with the same peripheral velocity of the work rolls and for different values of the peripheral velocity of the work rolls (introduction of contact asymmetry) aimed at obtaining a simple sheet metal after the rolling process.

The chemical composition was determined by the spectral method on the SPEKTROLAB B (Germany) device.

## 3. Research Results and Their Analysis

**Figure 6:**
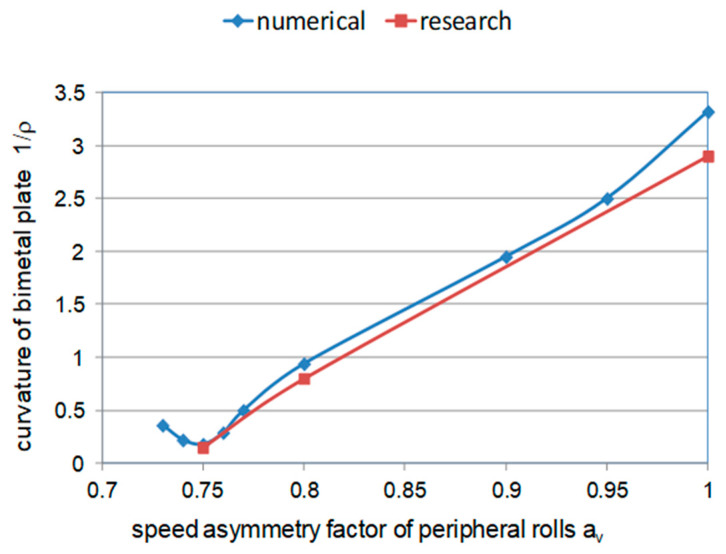
Relationship of the bimetallic sheet metal curvature on the kinetic asymmetry coefficient of the work rolls *a_v_* for the deformation ε = 0.18.

On the basis of numerical tests, the optimal value of the asymmetry coefficient (ASC) was determined, taking the bending of the bimetallic sheet metal as a determinant (Figure 6). The results of numerical tests were verified during laboratory tests and a high consistency was obtained for the optimal value of the asymmetry coefficient. For both numerical and laboratory tests, it was specified that the optimal value of the coefficient of asymmetry of the peripheral velocity of the work rolls is *a_v_* = 0.75. The results for two representative conditions (i.e., asymmetry *a_v_* = 0.75 (straight band) and *a_v_* = 1.0 (most bent strand)) are presented in a subsequent part of the paper.

The value of the ASC *a_v_* = 0.75 gave the possibility of obtaining a straight band. Later in the work, in order to determine the impact of the ASR process on the changes of microstructure occurring in the bimetal materials presented, laboratory tests for the asymmetry coefficients *a_v_* = 1.0 and the deformation ε = 0.18 were made as a comparison. The numerical tests of the curvature of bimetallic sheet metals after the traditional rolling process was 1/ρ = 3.33 [1/m], while as a result of applying the asymmetry with the rolls ASC *a_v_* = 0.75, it was 1/ ρ = 0.22 [1/m]. As a comparison of this parameter, measurements were made after physical rolling processes analogously conducted in laboratory conditions. It was found that this curvature is 2.94 [1/m] for *a_v_* = 1.0, while it is 0.15 [1/m] for *a_v_* = 0.75.

**Figure 7:**
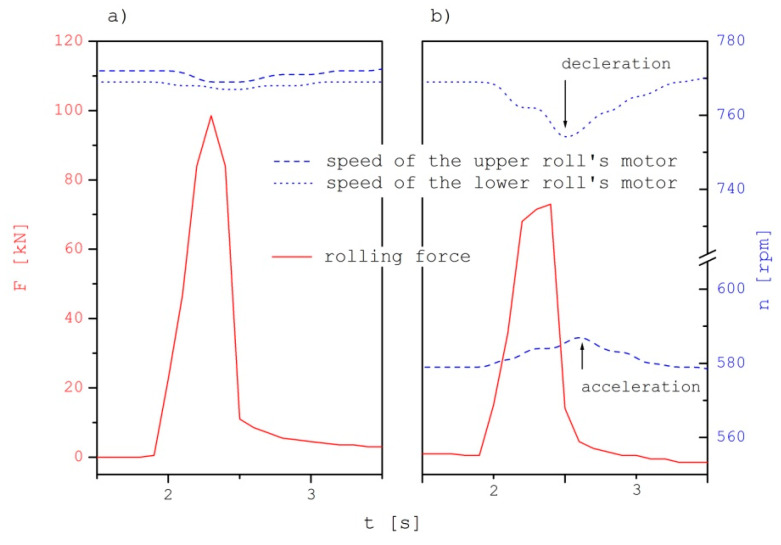
The course of changes in the value of the metal pressure force on the rollers and the rotational speed of the drive motors of the upper and lower rollers, (**a**) during symmetrical rolling, (**b**) optimal asymmetrical rolling.

During those tests, the pressing force of metal on the rolls was also measured. This was, made directly by force and rolling moment sensors. The indirectly measurement made on the basis of signals received from frequency converters supplying the driving motors of the upper and lower rollers. During the experimental tests, made with use of the DUO-300 rolling mill, two CL21 force transducers with a range of 250 kN, placed between the upper roll bearing cases and the adjusting screws, were used. The average force of metal pressure on the rolls during the symmetrical process was 89 kN, while it was significantly decreased by 20.2%, to the level of 71 kN, during the asymmetric rolling process. The courses of changes of these forces are shown in Figure 7. Described in the literature [1] the phenomenon of drop in the value of the metal pressure on the rolls is as resulting from the introduction of additional elongating stresses to the bimetallic rolling process on the material side with higher deformation resistance and additional compressive stresses on the material side with lower deformation resistance. As a result, it enables control of the deformation unevenness of the bimetallic layers as well as its curvature.

Then, on the basis of the numerical modeling and laboratory tests, a comparative analysis of the occurring structural changes during the rolling process was performed.

Figure 7 shows the average grain size distributions for the asymmetric rolling process of bimetallic sheet metals X2CrNiMo17-12-2 + 10CrMo9-10 obtained as a result of numerical calculations.

**Figure 8:**
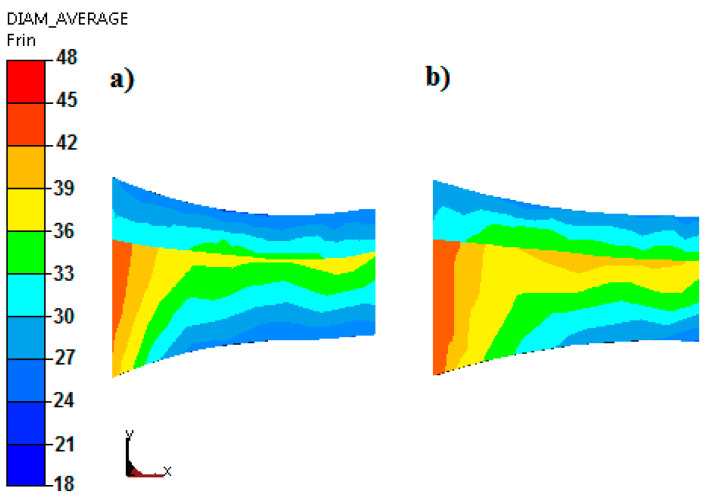
Average grain size distributions during rolling of bimetallic sheet metals X2CrNiMo17-12-2 + 10CrMo9-10 with relative deformation ε = 0.18 for: (**a**) *a_v_* = 0.75 and (**b**) *a_v_* = 1.0.

By analyzing the influence of the asymmetry of the peripheral velocity of the work rolls on the changes in their microstructure occurring in the deformed materials, this relationship was clearly shown. On the basis of the distributions of average grain size presented in Figure 8, it can be concluded that the introduction of asymmetry has a positive effect on microstructural changes. For the asymmetry of the peripheral speed of the work rolls (*a_v_* = 0.75, Figure 7b), lower average grain sizes were obtained for both layers of the bimetallic sheet metal.

The quantitative analysis of the grain size and microstructure components was performed using the comparative method, in accordance with the ISO standard and using the NisElements D program. The minimum number of N micrographs required to determine the component volume fraction with the assumed confidence level of 0.95 Uα and 0.1 absolute error of estimation *γ*, was determined on the basis of a “small sample” using the relationship (2) [38,39].

(2) $$N={\left(\frac{U_{\alpha }\cdot S}{\gamma \cdot \bar{x}}\right)}^{2}$$

where:

*N*—number of necessary measurements;

*Uα*—confidence level;

*S*—standard deviation;

*γ*—absolute error of estimation;

$\bar{x}$—average value.

The rolling process in laboratory conditions was conducted at a temperature of 1080 °C. The determination of the occurring structural changes was possible only as a result of the so-called structure freeze. The rolled samples were quickly cooled with water for this purpose. Then, samples were taken for microstructural tests and a comparative analysis of the state of the microstructure obtained after joining with the explosive welding method and after the symmetrical and asymmetric rolling process. Microstructural studies were performed using a Nikon MA-200 optical microscope.

**Figure 9:**
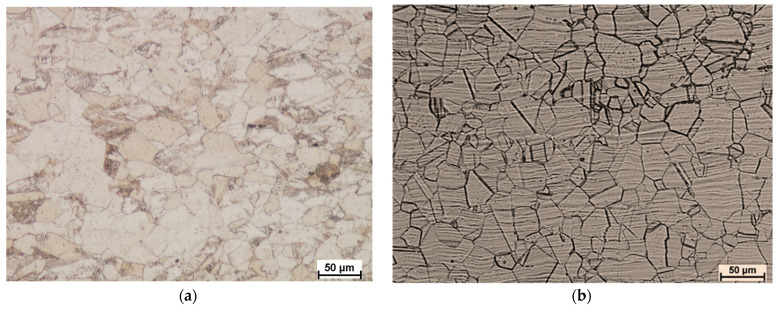
Microstructure of samples taken before rolling (**a**) X2CrNiMo17-12-2 steel (**b**) 10CrMo9-10 steel.

This section may be divided into subheadings. It should provide a concise and precise description of the experimental results, their interpretation, and the experimental conclusions that can be drawn. Figure 9 shows the microstructure of the starting material subjected to the rolling process.

**Figure 10:**
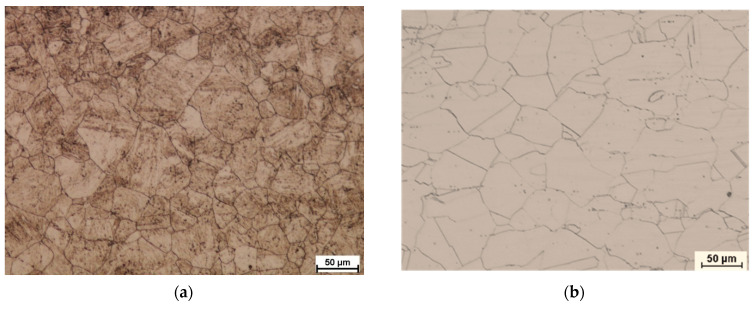

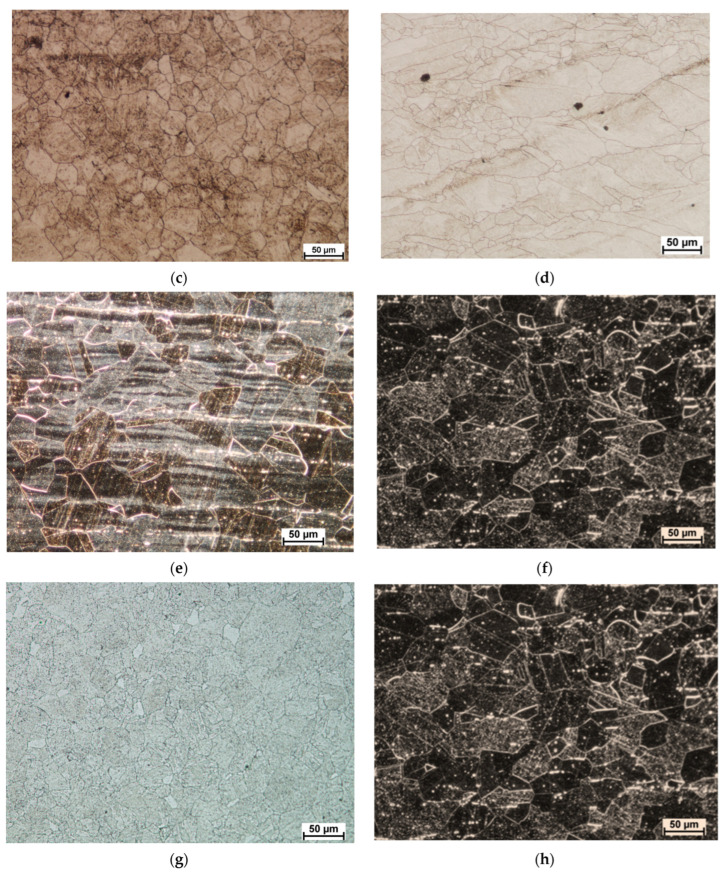
The microstructure of the samples taken for tests after rolling with a relative deformation ε = 0.18, Cooling variant I: (**a**) X2CrNiMo17-12-2 steel for *a_v_* = 1.0, (**b**) 10CrMo9-10 steel for *a_v_* = 1.0, (**c**) X2CrNiMo17-12-2 steel for *a_v_* = 0.75, (**d**) 10CrMo9-10 steel for *a_v_* = 0.75; Cooling variant II: (**e**) X2CrNiMo17-12-2 steel for *a_v_* = 1.0, (**f**) 10CrMo9-10 steel for *a_v_* = 1.0, (**g**) X2CrNiMo17-12-2 steel for *a_v_* = 0.75, and (**h**) 10CrMo9-10 steel for *a_v_* = 0.75.

In order to present the discussed problem of grain fragmentation as a result of the applied asymmetry of the peripheral velocity of the work rolls, Figure 10 shows the pictures of the microstructure of both components of the bimetallic sheet metal in the direct proximity of the joint area.

Figure 10 shows pictures of microstructures of materials forming the tested bimetal for the cooling variants I and II for the symmetrically and asymmetrically conducted rolling process. Then, a comparative grain size analysis was performed for both bimetal components. After the tests, it was found that during cooling in water for X2CrNiMo17-12-2 steel, the average grain size obtained during rolling for *a_v_* = 1.0 is 36 μm, and the structure is uneven (grain sizes from 20 μm to 50 μm are present). However, for a sample rolled with asymmetry *a_v_* = 0.75, the average grain size is 29 μm (where the grain size ranges from 20 μm to 40 μm). By making analogous observations for the component 10CrMo9-10, it was noted that the majority of grains are very similar in size (about 40 μm) and evenly distributed for *a_v_* = 1.0. On the other hand, after introducing the asymmetry in the peripheral velocity of the work rolls, a distinct local grain fragmentation is observed. The structure is uneven, with the majority of grain size from a few to 20 μm, and grain size of approx. 60 μm. After averaging (from 12 measurements where the 6 largest and 6 smallest representative grains were measured), the grain size was 22 μm. Considering the average value of the grain size, it is a positive influence on the structural changes. However, due to the properties of the material, it would be most advantageous if these grains had an even size.

**Figure 11:**
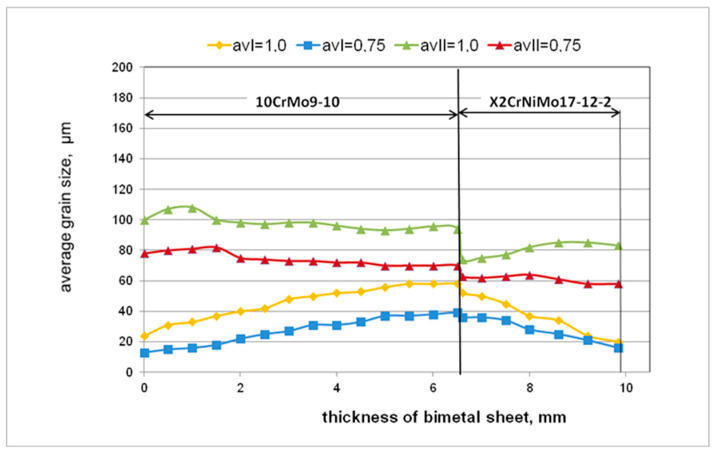
The distribution of the average grain size at the height of the bimetallic sheet metals for two cooling variants (I and II) of the samples after symmetrical *a_v_* = 1.0 and the asymmetric rolling process *a_v_* = 0.75.

The next stage of the research was to analyze the structural changes for the second cooling condition (i.e., slow cooling of the samples after rolling). The samples were rolled for analogous parameters of the rolling process, which enabled a comparative analysis of the structural changes taking place due to the implementation of the sample cooling method. The first variant shows the direct influence of kinetic asymmetry on the process of structural changes. The second variant of cooling reflects both the influence of kinetic asymmetry on the occurring phenomenon of recrystallization and grain growth after the rolling and cooling process. On the basis of the observations, it can be concluded that a clear grain growth occurs after the slow cooling of the samples. It is most visible in the external areas of the tested samples (i.e., at the surfaces of the external layers of Figure 11). On the other hand, based on the analysis of images for variant II of cooling presented in Figure 10e–h, an increase in the evenness of the grain distribution is clearly visible. When analyzing the grain size, it can be concluded that the applied kinetic asymmetry has a significant impact on this parameter. When observing the pictures presented in Figure 10e, it can be concluded that the average grain size obtained from the measurement of 12 representative grains, after rolling for *a_v_* = 0.75, ε = 18% and cooling in the air for the X2CrNiMo17-12-2 steel layer is 80 μm, while it is 91 μm 10CrMo9-10 for the steel layer. The average grain size obtained after rolling and cooling in the air for the X2CrNiMo17-12-2 layer is 60 μm for samples rolled with *a_v_* = 0.75 and ε = 18%, while it is 77 μm for the layer 10CrMo9-10. Particular attention should be paid to the fact that the vast majority of grains obtained after asymmetric rolling and cooling in the air are of similar size. By summarizing the analysis of the test results, it can be concluded that the asymmetry of the peripheral velocity of the work rolls aimed at obtaining straight sheet metals after the exit from the deformation, and the gap has a positive effect on grain fragmentation.

In order to confirm the obtained test results, the structural changes at the height of the bimetallic sheet metals after the rolling process were determined for *a_v_* = 1.0 and *a_v_* = 0.75. Samples and a specific average grain size were made for this purpose, as shown in Figure 11.

By analyzing the changes in the grain size of the materials constituting the bimetallic sheet metals tested according to the first cooling variant, it can be concluded that the asymmetry of the peripheral velocities of the work rolls has the greatest impact on the grain fragmentation of the 10CrMo9-10 layer. The differences in the average grain size reach almost 20 μm. These differences are much smaller for the X2CrNiMo17-12-2 layer, reaching 10 μm in the joint area to a few m at its edge (which is practically within the error limit). While analogously analyzing the structural changes at the height of the bimetallic sheet metals after cooling in the air, it is observed that the kinetic asymmetry has a positive effect on the grain fragmentation for these conditions. The differences in the average grain size at the edges of the sheet metals exceed the value of 20 μm for both layers, while closer to the joint area these differences are smaller. Despite the observed differences in the distribution of the average grain size at the height of the bimetallic sheet metals for both variants of cooling the samples after rolling, a positive effect of kinetic asymmetry on the grain size after the rolling process is observed for the value of *a_v_* = 0.75.

Average grain size distributions presented in Figure 11 were obtained for the same process temperature T = 1080 °C and relative deformation ε = 18%. The course of structural changes depends on the value of the kinetic asymmetry coefficient *a_v_*. A cooling variant was analyzed. Direct cooling in water was intended to show the effect of the asymmetry of the speed of the work rolls on the grain size distribution at the height of the sheet metal. On the other hand, the variant of cooling in air allows us to finally assess the effect of asymmetry on structural changes occurring in real conditions after the rolling process. On the basis of this research, it was observed that the asymmetry of the speed of the work rolls significantly contributed to the greater grain fragmentation. According to the authors of the study, this is related to the introduction of additional elongating stresses in the layer with higher deformation resistance (X2CrNiMo17-12-2) as a result of asymmetry and additional compressive stresses in the layer with lower deformation resistance (10CrMo9-10). As a result, higher evenness of the deformation distribution into the bimetallic sheet metal layers is observed, which in the end enables one to obtain a straight sheet metal after coming out the rolling gap.

**Figure 12:**
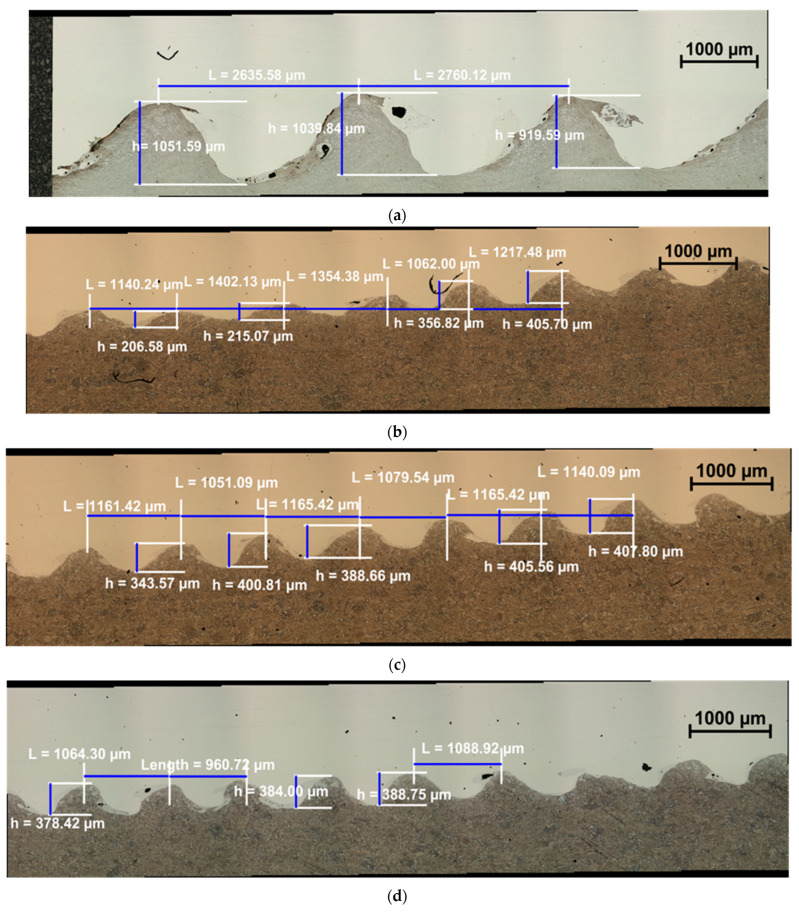
Microstructure of the joint area of X2CrNiMo17-12-2 + 10CrMo9-10 bimetallic sheet metals (**a**) bimetallic material after direct connection, (**b**) after asymmetric rolling for *a_v_* = 0.75, (**c**) after asymmetric rolling for *a_v_* = 0.8, and (**d**) after rolling for *a_v_* = 1.0.

The next stage of work was the analysis of the joint area of bimetallic sheet metals. The character of the joint of bimetallic sheet metals and the changes taking place in the joint area as a result of hot plastic forming were analyzed. The observations were aimed at showing how the melting produced during the welding with the explosive method affects the character of changes in the joint area and its durability [27,28,29]. The samples for metallographic tests were made on the side surface of the samples subjected to rolling and then cooling. Figure 12a–d presents the microstructures of the joint area, which show the influence of kinematic asymmetry on the characteristics of the joint area.

As can be seen, the area of the joint changes in the initial state and after rolling. Table 2 shows the average values of the geometrical parameters of the waves in the area of the connection of bimetallic sheet metals for the starting material and those subjected to plastic shaping.

It should be noted that the corrugated connection obtained as a result of explosion welding is maintained in each of the tested variants. Based on the observations made in Figure 12a–d, it can be stated that the share of bimetallic sheet metals formed during the joining process has not changed. However, as a result of plastic shaping, the peak heights are significantly reduced by about 70–61%. Likewise, the average peak wave distance is also reduced from about 62 to 54%. This is important since this type of connection, which increases the surface of interaction, is removed after the plastic working process in most cases [27,28,29]. Naturally, from the point of view of the production of semi-finished products for further rolling, for example, a completely linear connection is ideal. However, it also has its limitations. Considered to be the stronger connection, it is corrugated, and the reconstruction of this area during hot rolling, while maintaining the character, guarantees high properties in the joint area, which predestines the material for more severe working conditions.

**Figure 13:**
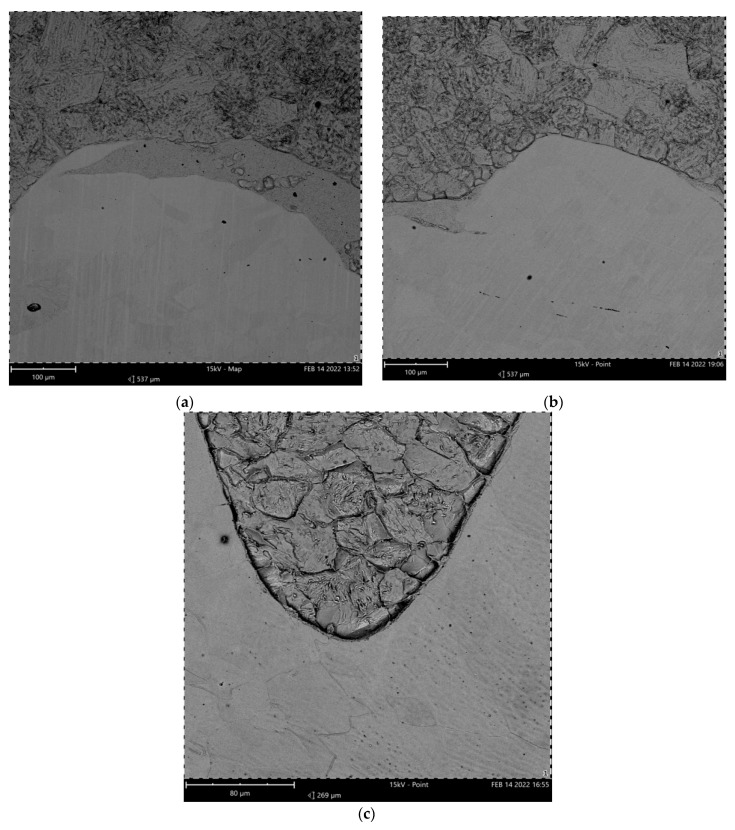
SEM microstructure of the joint area samples taken (**a**) after rolling for *a_v_* = 1.0, (**b**) after asymmetric rolling for *a_v_* = 0.75, and (**c**) after asymmetric rolling for *a_v_* = 0.8.

**Figure 14:**
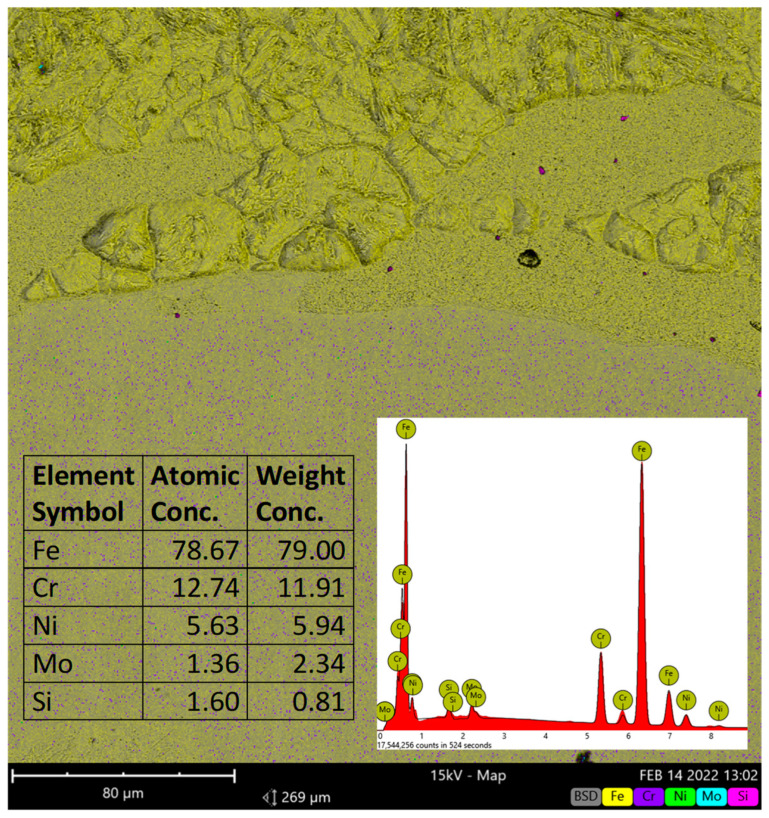
The chemical composition of the joint area (sample after direct connection).

In order to deepen the analysis of the microstructure and to assess the chemical composition of the joint area, an analysis using SEM microscopy was also performed. A Phenom XL scanning microscope was used, and the obtained results are presented on Figure 13. The chemical composition of the joint area is show on Figure 14. The analysis was made for the sample after direct connection since this does not change during plastic deformation.

The analysis of the joint area made with the use of the SEM microscopy confirmed that for all samples there are no observed cracks. According to the obtained results it can be stated that the process parameters can guarantee not only straight bimetalic plates but also plates with good quality of joints. It also can be seen that hot deformation provides the possibility for recrystallisation in both layers as well as in the joint area. This is especially important in the aspect of the presence of intermetallic layers occurring during explosive welding. The chemical composition presented in the Figure 14 confirmed the presence of all main elements.

## 4. Conclusions

The paper presents the results of a comprehensive study of the influence of the asymmetric rolling process of bimetallic sheet metals, both on the shape of the band after leaving the deformation gap and on microstructural changes. To highlight this issue, the process was conducted for two variants:

- Cooling in water in order to present the effect of asymmetry on structural changes immediately after the rolling process,
- Slow cooling in the air, which was aimed at presenting the effect of the asymmetry of the speed of the work rolls on the occurring microstructural changes in the finished product (i.e., bimetallic sheet metal).

The research results presented in the paper confirm the fact that the introduction of different peripheral velocities of the work rolls contributes to the improvement of the quality of the band shape. However, what was the main topic of the work and what is the scientific achievement of the presented considerations is the statement of a significant influence on the microstructural changes occurring during the asymmetric rolling process. It was observed that not only a straight sheet metal is obtained for the value of *a_v_* = 0.75, but also a sheet metal of a more even and smaller grain size. This phenomenon will probably have a direct impact on the increase of the strength properties of the materials constituting the bimetallic sheet metal. To sum up, it can be stated that the use of asymmetry of the peripheral velocities of the work rolls has a beneficial effect on the ongoing structural changes. An important effect that was obtained in the work is the effect of maintaining a wavy connection in the zone of the bimetallic joint. The wavy joint area typical of explosive welding is annihilated by plastic deformation and turns into a flat joint in most cases. The used parameters enabled the maintenance of an advantageous type of connection. Visible, typical for the method of explosive remelting, in the initial state during the deformation of the microstructure, was blurred during the recrystallization processes. After the phenomena of recrystallization, which resulted in significant fragmentation of the microstructure, the wavy joint area was also remade. However, what should be emphasized is that the strongly developed, positive character of the joint zone has not been lost from the point of view of the mechanical properties of the tested material.

## Figures and Tables

**Table 1:** Chemical composition of the tested materials 10CrMo9-10 *and* X2CrNiMo17-12-2.

Materials	Chemical Composition, Mass Percent											
	C	Mn	Cr	Mo	Ni	Al	Cu	Co	P	Si	S	Fe
10CrMo9-10	0.12	0.52	2.0	0.91	-	0.03	-	-	0.01	0.35	0.01	rest
X2CrNiMo 17-12-2	0.03	2.0	16.61	2.0	10	-	0.34	0.25	0.04	0.75	0.01	rest

**Table 2:** Geometric parameters of the joint area of bimetallic sheet metals X2CrNiMo17-12-2 + 10CrMo9-10.

Designation	Average Wave Height, µm	Average Peak Distance, µm
Initial state	1003.67	2697.85
*a_v_* = 0.75	296.04	1235.25
*a_v_* = 0.80	389.28	1127.16
*a_v_* = 1.0	383.72	1037.98

## Data Availability

Data sharing is not applicable to this article.
